# The dynamic association between family functioning and externalizing problems in adolescents from low-income families: a latent change score model study

**DOI:** 10.3389/fpsyg.2026.1768618

**Published:** 2026-04-24

**Authors:** Zhugui Liang, ShiWei Tang, ZhiHua Li

**Affiliations:** 1 School of Physical Education and Sport Science, Hengyang Normal University, Hengyang, China; 2 School of Education, Hunan University of Science and Technology, Xiangtan, China

**Keywords:** dynamic association, externalizing problems, family functioning, latent change score model, low-income youth

## Abstract

Based on family systems theory, this study examines the longitudinal dynamic relationship between family functioning and externalizing problems among adolescents from low-income families. Using the Adolescent Family Growth and Recovery Assessment (APGRA) and the Strengths and Difficulties Questionnaire (SDQ), 468 adolescents from low-income families were tracked four times over a 2-years period, and the data were analyzed using a latent class scalar model (LCSM). The results showed that: (1) family functioning and externalizing problems among adolescents from low-income families were significantly negatively correlated; family functioning declined significantly over time (μ_XS_ = −10.45, *p* < 0.01), while externalizing problems increased significantly over time (μ_γS_ = 9.90, *p* < 0.05); (2) changes in family functioning and externalizing problems exhibited a bidirectional negative temporal association: levels of externalizing problems positively predicted subsequent changes in family functioning (γ_Xγ_ = 0.45, *p* < 0.001), changes in externalizing problems negatively predicted changes in family functioning (ξ_Xγ_ = −0.37, *p* < 0.01), and changes in family functioning negatively predicted changes in externalizing problems (ξ_γX_ = −0.46, *p* < 0.01), supporting the reciprocal effects model. In summary, there is a reciprocal dynamic relationship between family functioning and externalizing problems among adolescents from low-income families. This study provides longitudinal empirical evidence for the prevention and intervention of problem behaviors in this population.

## 1 Introduction

Low-income households refer to impoverished households that were registered in the database during the targeted poverty alleviation period based on local standards; during the survey period, the criteria for rural areas defined a low-income household as one with a per capita net income of less than 3,026 yuan per year (Liu and Xu, 2017). Poverty does not merely signify a lack of economic resources; it is also accompanied by structural challenges such as insufficient investment in family education, heavy caregiving burdens, frequent life stressors, and weak social support. Together, these factors create a high-risk environment for adolescent development, making psychological and behavioral issues among adolescents from low-income families particularly worthy of close attention. The 2025 Global Multidimensional Poverty Index report released by the United Nations Development Programme reveals a grim reality (OPHI and UNDP, 2025): among 6.3 billion people across 109 countries, over 18.3% (approximately 1.1 billion individuals) live in severe multidimensional poverty. Children are disproportionately affected, with those under 18 constituting half of the global multidimensional poor population–reaching 584 million. The majority of the world’s poor reside in rural areas, accounting for a staggering 83.5% of the total. Economic adversity imposes numerous negative impacts, particularly on adolescents. Research indicates that children raised in economically disadvantaged households exhibit developmental disparities compared to their peers across multiple dimensions, including but not limited to delayed physical development (Hanson et al., 2013), impaired cognitive abilities (Chen and Zhang, 2024), lower academic achievement (Baker et al., 2012), weaker self-regulation skills and more severe emotional disorders (Flouri et al., 2014), as well as higher risks of problem behaviors (Gutman et al., 2019). It is worth noting that, compared to internalizing problems, children who face prolonged economic hardship are more significantly affected by externalizing problems, which are more overt and socially disruptive, more likely to attract the attention and intervention of the family system, and have a higher prevalence during adolescence than internalizing problems (Comeau and Boyle, 2018). Therefore, in-depth research and understanding of these externalizing problems are crucial for promoting their holistic development.

Externalizing problems manifest as maladaptive behavioral traits such as conflict with others, defiance, aggression, rule-breaking, emotional dysregulation, and hyperactivity; they encompass a range of behavioral presentations, including conduct disorder, attention-deficit/hyperactivity disorder, and oppositional defiant disorder (Goodman et al., 2010). Adolescence is a peak period for externalizing problems. Research indicates that some children exhibit persistent and high-intensity externalizing problems as early as early childhood (before age 10) (Moffitt, 1993; Bradley, 2007), with these behaviors potentially evolving into antisocial conduct during adolescence and adulthood (Broidy et al., 2003). Adolescence is characterized by asynchronous physical and psychological development, placing individuals in a state of disequilibrium and confronting them with unique conflicts and psychological crises. During this phase, heightened self-awareness combined with neuropsychological deficits, destructive social relationships, poor parenting practices, and other stressors increases susceptibility to inattention, social difficulties, hyperactivity, and impulsive behaviors (Zhang et al., 2022). Therefore, this study focuses on the developmental trajectories of externalizing problems among adolescents from low-income families, aiming to provide more targeted support and intervention strategies to enhance their social adaptation capabilities.

Family functioning serves as a crucial indicator for assessing the quality of the home environment, parent-child relationships, and parental relationships. It encompasses five key dimensions: family adaptability, cooperation, growth, emotional expression, and intimacy. Research has shown that dysfunctional families increase the risk of externalized problems such as smartphone dependence (Shi et al., 2017), self-harm behavior (Hammond et al., 2025), and bullying behavior (Ma et al., 2024) among adolescents (Wang et al., 2024). For low-income families, multiple adversities–such as financial pressures, caregiving burdens, and life uncertainties–are more likely to undermine family cohesion and problem-solving abilities, resulting in relatively weaker family structures and functions, which in turn increases the risk of behavioral problems among adolescents (Banovcinova et al., 2014). According to family systems theory, interactions among family members profoundly influence each other. The strength of family functioning directly shapes adolescents’ behavioral patterns, while their developmental changes also impact the entire family system (Bowen, 1974). When viewed through the lens of family systems theory in the context of poverty, economic pressures are not merely external factors but rather ongoing stressors that act upon the family system. These pressures directly alter family members’ interaction patterns, emotional bonds, and problem-solving efficiency, thereby influencing the dynamic relationship between family functioning and adolescents’ externalizing problems. Thus, the relationship between adolescent family functioning and externalizing problems may manifest through three distinct models: the family effects model, the child effects model, and the reciprocal effects model (Wang et al., 2022).

The family effects model emphasizes the holistic influence of the family unit, examining the long-term impact of factors like family environment, structure, and interactions on adolescent psychological and behavioral development (Wood et al., 2021). Research indicates that the quality of family relationships influences adolescents’ externalizing problems (Jensen and Lippold, 2018). Deficiencies in family functioning and increased feelings of loneliness may weaken an individual’s behavioral control and learning efficacy, thereby triggering externalizing problems (Lu, 2020). Adolescents from low-income families with lower levels of familial care exhibit higher rates of delinquent behavior (Shek and Lin, 2016). The child effect model focuses on how children’s traits and behaviors reciprocally influence the family environment and parental behavior. For instance, children’s externalizing problems (such as aggression or defiance) may affect parenting styles and family atmosphere (Gross et al., 2008), disrupting communication and intensifying family stress. The reciprocal effect model emphasizes bidirectional influences among family members, where the family environment and children’s behaviors interact to mutually shape each other’s development. This model often examines reciprocal relationships through cross-temporal analyses, investigating the dynamic changes in family environment and child behavior over time (Deng et al., 2022). In the context of low-income families, the dynamic interactive mechanisms revealed by family systems theory are particularly pronounced. Economic stress, as a chronic source of stress, directly undermines family functioning by compromising parents’ mental health and exacerbating marital conflict, making the family system more susceptible to falling into a cycle of dysfunction (Conger et al., 1994; Ponnet et al., 2015). In this context, the predictive role of family functioning on adolescents’ externalizing problems (the family effect) may be more pronounced; simultaneously, adolescents’ externalizing problems (such as aggression and defiant behavior) may further exacerbate already fragile family functioning by depleting family resources and disrupting the family atmosphere (the child effect) (Miller et al., 2024; Xu et al., 2025). Consequently, a high-intensity, bidirectional vicious cycle is more likely to emerge between family functioning and adolescent behavior in low-income families, making the reciprocal effects model a central theoretical framework for exploring the mechanisms underlying problems in this population.

Currently, longitudinal research on the relationship between adolescent family functioning and externalizing problems remains limited. Domestically, only the family effects model (Hu et al., 2009) and child effects model (Wang et al., 2022) have been partially validated. Existing international research has employed cross-lagged analyses of parental psychological control and internalizing/externalizing problems among high school students (Kaniousonyte and Zukauskiene, 2016), revealing reciprocal influences between these factors. However, there has been relatively little discussion of the family reciprocity model, and there remains a lack of sufficient longitudinal evidence regarding whether there is a dynamic, mutually predictive relationship between family functioning and externalizing problems, particularly among low-income families–a high-risk group.

Therefore, deepening our understanding of the dynamic relationship between family functioning and externalizing problems among adolescents in economic adversity holds profound significance for preventing and reducing externalizing problems while promoting their comprehensive healthy development.

Traditional longitudinal studies primarily rely on covariance analysis, such as cross-lagged regression models. While these models have validated reciprocal effects, they fail to capture the rates of change or the bidirectional dynamic processes, and do not adequately account for structural changes in the mean levels of variables. As a result, the dynamic changes in variables over time have been overlooked in past research (Kaniusonyte and Zukauskiene, 2016). Latent Change Score Modeling (LCSM) provides a complementary analytical framework that not only combines the strengths of cross-lagged regression–allowing for the inference of longitudinal predictive relationships among variables–but also enables the modeling of latent longitudinal changes in the mean levels of study constructs (McArdle, 2009). LCSM exhibits several significant advantages over cross-lagged regression models. First, it effectively controls for measurement error arising from observed scores by utilizing latent variables, thereby enhancing research accuracy (Alexandra et al., 2021). Second, LCSM captures the dynamic interaction between family functioning and externalizing problems over time. Within the LCSM framework, when changes in family functioning over time trigger subsequent shifts in externalizing problems, family functioning can be regarded as a prospective indicator of externalizing problems, while externalizing problems serve as a lagging indicator of family functioning. If this predictive relationship exhibits bidirectionality–i.e., reciprocity–it indicates mutual predictive effects between the two variables. Specifically, if family functioning and externalizing problems mutually predict each other over time, and the path of family functioning influencing externalizing problems is more significant than the reverse path of externalizing problems affecting family functioning, we can infer that although changes in family functioning and externalizing problems are associated, changes in externalizing problems actually occur after changes in family functioning–that is, a lag exists. Therefore, compared to cross-lagged regression models, the LCSM model may offer a more precise and flexible analytical tool for examining the longitudinal dynamic relationship between family functioning and externalizing problems (Gao et al., 2021).

In summary, this study proposes to use a latent difference score model to investigate the dynamic relationship and interaction patterns between family functioning and externalizing problems among adolescents from low-income families, and positing the following hypotheses: (1) family functioning and externalizing problems among adolescents from low-income families undergo dynamic changes over time. (2) A bidirectional predictive relationship exists between family functioning and externalizing problems among these adolescents, supporting the reciprocal effects model.

## 2 Methods

### 2.1 Participants

This study employed cluster random sampling to select 14 primary and secondary schools in Hunan Province, conducting four follow-up surveys over 2 years targeting students from low-income families. Low-income families refer to those registered as impoverished households during the targeted poverty alleviation period based on local standards. The rural poverty threshold during the survey period was defined as a per capita net income below 3,026 yuan per year. While this standard is highly region-specific, it also has certain limitations. The specific survey timepoints were as follows: October 2022 (T1), 598 samples; June 2023 (T2), 574 samples; February 2024 (T3), 542 samples; October 2024 (T4), 584 samples. Some data became invalid due to student illness, withdrawal, transfer, or lack of diligence in completing questionnaires. After excluding irrelevant data, 468 valid samples from participants who completed all four rounds of the survey were retained, resulting in an overall retention rate of 78.3%. Among the valid samples, 174 were male (37.2%) and 294 were female (62.8%); 109 were elementary school students (23.3%), 259 were middle school students (55.3%), and 100 were high school students (21.4%). In the first survey, the average age of participants was 12.62 years (SD = 2.21). To examine whether sample attrition caused selection bias, we conducted an attrition analysis comparing participants who fully participated in all four surveys (*n* = 468) with those excluded due to attrition or invalid data (*n* = 130) on key variables at T1, including gender, age, household income, family functioning, and externalizing problems. The results showed no significant differences between the two groups on any of the variables (*p* > 0.05), indicating that attrition did not cause systematic bias. For missing data, this study employed full-information maximum likelihood estimation (FIML), a method that uses all available data points for parameter estimation and provides consistent and valid estimates under the assumption of missing at random (MAR).

#### 2.1.1 Selection of research participants and data processing procedures

(1) Sampling: using cluster random sampling, 14 primary and secondary schools were randomly selected from across Hunan Province to serve as pilot sites for the survey. The study participants were defined as students from low-income families at these schools (specifically, families registered as impoverished during the targeted poverty alleviation period, with a per capita net income in rural areas below 3,026 yuan per year).
(2) Implementation of longitudinal survey: questionnaires were distributed and collected at four specific time points: October 2022 (T1), June 2023 (T2), February 2024 (T3), and October 2024 (T4). During each survey, trained interviewers provided on-site guidance to students to ensure the questionnaires were completed correctly.
(3) Sample screening: after collecting the questionnaires, invalid samples were excluded based on the following criteria: students unable to participate in subsequent surveys due to illness, leave of absence, or transfer; and those who submitted incomplete questionnaires or demonstrated a lack of seriousness.
(4) Determination of valid samples: data from the four surveys were aggregated, retaining only samples that participated in all four surveys. Demographic information and the sample retention rate for the valid samples were calculated.
(5) Testing for selection bias: using attrition analysis, we compared key variables–such as gender, age, family income, family functioning, and externalizing problems–between participants who completed the full study (*n* = 468) and those who were excluded (*n* = 130) at T1. We used these comparisons to determine whether systematic bias existed.
(6) Missing data handling: Full Information Maximum Likelihood (FIML) estimation was used to handle missing values in the data. Parameter estimates were derived using all available data points to ensure the consistency and validity of the results.

### 2.2 Measures

#### 2.2.1 Externalizing Problems Scale

This study used the total scores of the Conduct Problems and Hyperactivity factors from the Strengths and Difficulties Questionnaire (SDQ) to assess externalizing problems in adolescents. These two factors consist of 10 items, each scored on a 3-point scale; higher scores indicate a higher level of externalizing problems (Goodman et al., 1998; Roman et al., 2016). However, the Cronbach’s α coefficients for this scale across the four measurements were 0.63, 0.66, 0.66, and 0.68, respectively, indicating reliability below the generally accepted standard of 0.70. This constitutes a methodological limitation of this study. Low reliability may lead to increased measurement error, thereby underestimating the true strength of the association between variables to some extent or affecting the stability of parameter estimates. Future studies will use externalizing problem measurement tools with higher reliability (such as the Achenbach Child Behavior Checklist) or adopt a multitrait-multimethod design to correct for measurement error, further validating the reliability of the findings in this study.

##### 2.2.1.1 Procedures for the Externalizing Problems Scale

(1) Scale selection and determination: The Conduct Problems and Hyperactivity factors from the SDQ questionnaire were selected as the measurement tools for externalizing problems. A total of 10 items from these two factors were extracted, and the item wording and scoring rules were clarified.
(2) Standardization of scoring criteria: a 3-point scoring scale was adopted, with clear definitions for each score (e.g., “1 point = does not apply,” “2 points = partially applies,” “3 points = fully applies”) to ensure consistent understanding among all investigators and participants.
(3) Scale administration: the scale was distributed to participants during all four follow-up surveys. Participants completed the scale independently based on their actual circumstances, with survey administrators available on-site to answer questions and prevent completion bias.
(4) Reliability testing: after collecting scale data from the four measurements, statistical software was used to calculate Cronbach’s α for each measurement to assess scale reliability. Potential causes and implications of low reliability were also analyzed.

#### 2.2.2 Family Functioning Scale

Family functioning was assessed using the Family Care Index Scale (APGRA) (Takenaka and Ban, 2016), comprising 5 items representing 5 factors: adaptability, cooperation, growth, emotionality, and intimacy. Each item corresponds to one factor. A 3-point scoring system was applied: “Always true” = 3 points, “Sometimes true” = 2 points, “Rarely true” = 1 point. Higher total scores indicate better family functioning. A total score of 11–15 indicates good family functioning, 6–10 indicates moderate impairment, and 1–5 indicates severe impairment. In this study, the Cronbach’s α coefficients for the four administrations of the Family Functioning Scale were 0.79, 0.75, 0.80, and 0.79, respectively.

##### 2.2.2.1 Procedures for administering the Family Functioning Scale

(1) Scale selection: the APGRA Short Form was selected as the instrument for measuring family functioning. It consists of five items corresponding to five factors: adaptability, cooperation, growth, emotionality, and intimacy.
(2) Clarification of scoring rules: a uniform 3-point scoring standard was established, defining “frequently = 3 points,” “sometimes = 2 points,” and “rarely = 1 point.” Additionally, the total score grading criteria were clarified (11–15 points = good, 6–10 points = moderate impairment, 1–5 points = severe impairment).
(3) Administration of the scale: the scale was distributed concurrently with the Externalizing Problems Scale. During the four follow-up surveys, participants completed the scale based on their actual family circumstances, with on-site guidance from survey administrators to ensure accurate and standardized completion.
(4) Reliability validation: after collecting data from the four measurements, Cronbach’s α coefficients were calculated for each measurement using statistical software to verify the scale’s reliability within the study sample and confirm its applicability.

### 2.3 Data analysis

This study adopted an extension of Grimm et al.’s (2012) Latent Change Score (LCS) framework, employing the Latent Change Score Modeling (LCSM) in Mplus 8.0 to test hypotheses. The research examined whether changes in family functioning were associated with subsequent increases or decreases in externalizing problems, and whether increases in externalizing problems were linked to subsequent changes in family functioning. During data fitting, *i*_*x*_ (intercept) and *i*_*y*_ (intercept) were set as the true scores for family functioning and externalizing problems at T1, respectively. The path coefficient estimates for α_*x*_ and α_*y*_ were fixed at 1. Estimates for β_*x*_ and β_*y*_, ϕ_*x*_ and ϕ_*y*_, γ_*yx*_ and γ_*xy*_, ξ_*yx*_ and ξ_*xy*_, σ_*ex*_^2^ and σ_*ey*_^2^ were set equal across all time points (Grimm et al., 2012). Subsequently, changes in externalizing problems at the next time point (e.g., ΔExternalizing Problems, T2-T3) were predicted using changes in family functioning across the preceding two time points (e.g., ΔFamily Functioning, T1-T2). During model estimation, we controlled for individual differences in gender, age, family functioning, and externalizing problems.

#### 2.3.1 Data analysis procedures

(1) Data preparation: valid sample data collected from the four follow-up surveys (scores on the Externalizing Problems Scale and the Family Functioning Scale, gender, age, etc.) were organized; data accuracy was verified; outliers were removed; data formats were standardized; and the data were imported into Mplus 8.0.
(2) Determination of analysis method: a Latent Change Score Modeling (LCSM) was employed for hypothesis testing, based on the Latent Change Score (LCS) framework extended by Grimm et al. (2012) to clarify the research hypothesis (the mutual influence between changes in family functioning and subsequent changes in externalizing problems).
(3) Model parameter specification: model parameters were specified in Mplus 8.0. ξ1 (intercept) was set to the true score of family functioning at T1, and ξ2 (intercept) was set to the true score of externalizing problems at T1; the path coefficient estimates for α_*x*_ and α_*y*_ were fixed at 1; the estimates for β_*x*_, β_*y*_, ϕ_*x*_, ϕ_*y*_, γ_*yx*_, γ_*xy*_, ξ_*yx*_, ξ_*xy*_, σ_*ex*_^2^, and σ_*ey*_^2^ were set to be equal across all time points.
(4) Model fitting and testing: run the latent change score model to predict changes in externalizing problems at the next time point (e.g., ΔExternalizing Problems at T2-T3) based on changes in family functioning at the first two time points (e.g., ΔFamily Functioning at T1-T2), and test the model fit.
(5) Control of interfering variables: during model estimation, gender and age were included as control variables to account for their effects on individual differences in family functioning and externalizing problems.
(6) Results analysis: based on the model output, the interrelationship between changes in family functioning and changes in externalizing problems was analyzed to test the research hypotheses. The results were organized and interpreted.

## 3 Results

### 3.1 Descriptive statistics

The correlation coefficients and mean scores for family functioning and externalizing problems across the four time points are presented in Table 1. Results indicate that adolescents’ family functioning scores showed a significant negative correlation with externalizing problems across all four assessments, with correlation coefficients ranging from −0.15 to −0.38 (*p* < 0.001). Both the concurrent and sequential correlations between the two variables were significant. Concurrent correlation coefficients ranged from −0.28 to −0.38 (*p* < 0.001), while sequential correlation coefficients ranged from −0.15 to −0.29 (*p* < 0.001).

**TABLE 1:** Mean, standard deviation, and correlation matrix for family functioning and adolescent externalizing problems at four measurement time points.

Research variables	1	2	3	4	5	6	7	8
1. Family function T1	1							
2. Family function T2	0.41^***^	1						
3. Family function T3	0.29^***^	0.38^***^	1					
4. Family function T4	0.20^***^	0.35^***^	0.41^***^	1				
5. Externalization issues T1	−0.38^***^	−0.28^***^	−0.22^***^	−0.15^**^	1			
6. Externalization issues T2	−0.29^***^	−0.28^***^	−0.30^***^	−0.17^***^	0.54^***^	1		
7. Externalization issues T3	−0.17^***^	−0.17^***^	−0.36^***^	−0.18^***^	0.33^***^	0.41^***^	1	
8. Externalization issues T4	−0.19^***^	−0.17^***^	−0.25^***^	−0.28^***^	0.36^***^	0.47^***^	0.37^***^	1
*M*	11.56	11.26	11.11	11.22	15.47	15.55	16.10	15.78
SD	2.33	2.20	2.28	2.27	3.00	2.98	3.07	3.11

T , time . ^**^p < 0.01,

^***^p < 0.001.

### 3.2 LCSM model of family functioning and adolescent externalizing problems

#### 3.2.1 Test of measurement invariance

Prior to conducting latent change factor model analysis, this study first tested the measurement invariance of family functioning and externalizing problems across the four time points. Using multi-group confirmatory factor analysis, we sequentially tested configural invariance (structural invariance), metric invariance (factor load invariance), and scalar invariance (intercept invariance). The results showed that the change in CFI (ΔCFI) for family functioning across the four time points was −0.008 during the transition from configural invariance to metric invariance and −0.011 during the transition from metric invariance to scalar invariance. Neither value exceeded the critical threshold of 0.01, indicating that family functioning met the criteria for scalar invariance. For externalizing problems, the ΔCFI across the four time points was −0.006 during the transition from configural invariance to metric invariance and −0.009 during the transition from metric invariance to scalar invariance, also meeting the criteria for scalar invariance. These results indicate that the meanings of the two variables across the four measurements are comparable over time, providing a foundation for subsequent LCSM analysis.

#### 3.2.2 Results of the latent fractional model

**FIGURE 1:**
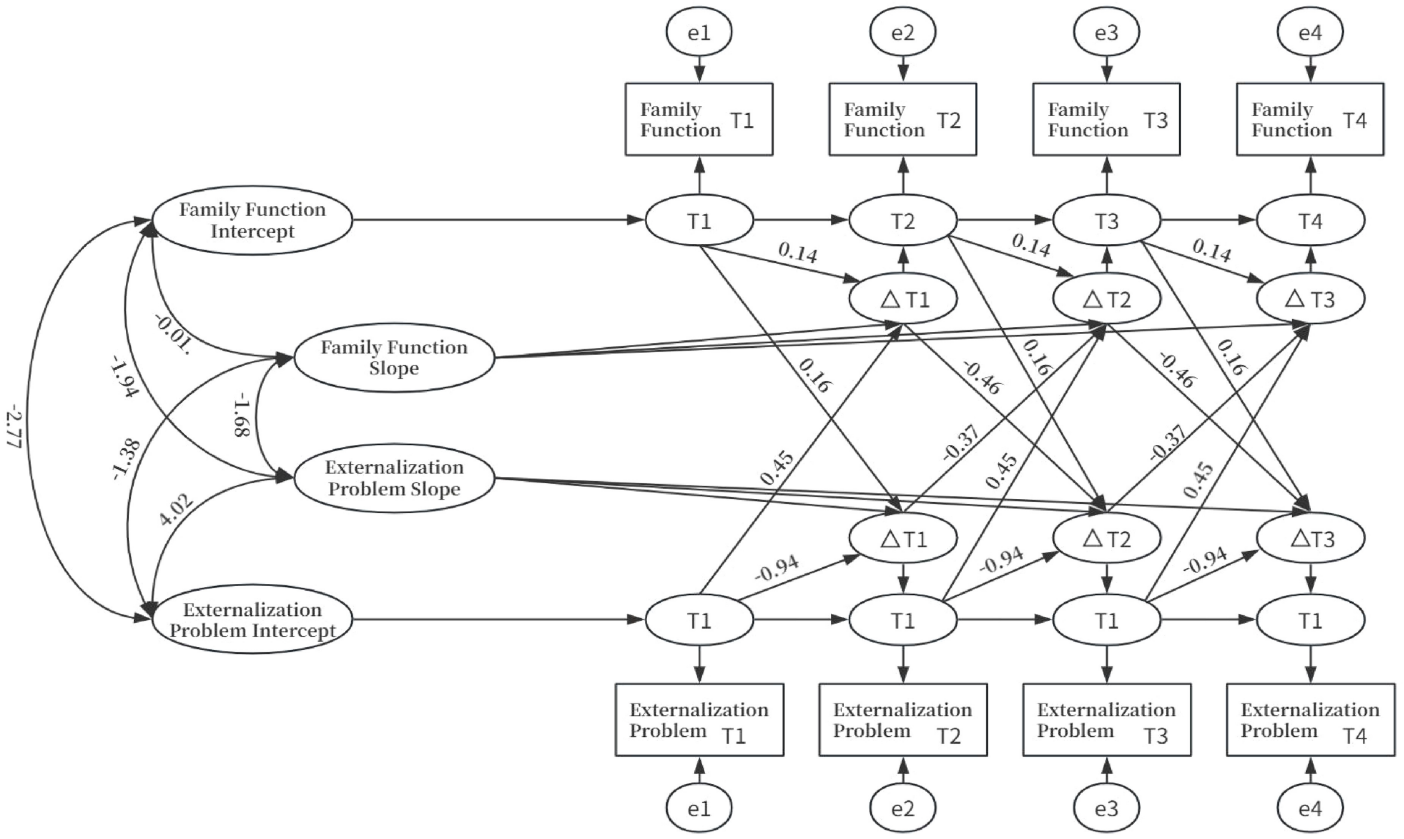
Latent Change Score Modeling (LCSM) diagram of family functioning and externalizing problems.

The LCSM model results are shown in Figure 1. This model examines the bidirectional influence pathways (ξ1, ξ2) between changes in family functioning scores among adolescents from low-income families and subsequent changes in externalizing problems. The overall model fit indices were good: χ^2^/df = 3.11, *p* < 0.001; CFI = 0.95; TLI = 0.93; RMSEA = 0.06 (90% CI = [0.05, 0.09]); SRMR = 0.07. The mean of the slope factors for family functioning is negative (μ_xs_ = −10.45, *p* < 0.01), indicating that the level of family functioning among adolescents shows a downward trend over time; the proportional change parameter (β_x_ = 0.14, *p* = 0.58) is not significant, suggesting that changes in the level of family functioning at the previous time point do not significantly influence the rate of subsequent change; the autoregressive effect of latent change scores for family functioning (ϕ_x_ = −0.12, *p* = 0.35) was not significant, indicating that changes in previous levels of family functioning did not significantly influence subsequent changes; the effect of externalizing problem levels on changes in family functioning (γ_xy_ = 0.45, *p* < 0.001) was significant, indicating that the higher the level of externalizing problems at a previous time point, the faster family functioning increased at subsequent time points. This result runs counter to the theoretically expected negative correlation, which may suggest that model specifications or data distributions require further examination. The effect of changes in externalizing problems on changes in family functioning (ξ_xy_ = −0.37, *p* < 0.01) was significant, indicating that a reduction in adolescents’ externalizing problems promotes an increase in family functioning.

The mean of the slope factor for externalizing problems was positive (μ_ys_ = 9.90, *p* < 0.05), indicating an upward trend in adolescents’ externalizing problems over time; The proportional change parameter was negative (β_y_ = −0.94, *p* < 0.001), indicating that higher levels of externalizing problems at an earlier time point were associated with slower increases in externalizing problems at subsequent time points. The autoregressive effect of latent change scores for adolescent externalizing problems was not significant (ϕ_x_ = 0.00, *p* = 0.99), suggesting that changes in externalizing problems at an earlier time point did not significantly influence subsequent changes. The effect of family functioning levels on externalizing problem changes was not significant (γ_yx_ = 0.16, *p* = 0.35), indicating that higher or lower family functioning at an earlier time point did not significantly influence the rate of increase in externalizing problems at subsequent time points; The effect of changes in family functioning on externalizing problem changes was significant (ξ_yx_ = −0.46, *p* < 0.01), indicating that improvements in family functioning among adolescents promote reductions in externalizing problems.

## 4 Discussion

This study employed a 2-years, four-wave longitudinal design and utilized latent class regression to examine the dynamic associations and interaction patterns between family functioning and externalizing problems among adolescents from low-income families. The results indicate that family functioning declined over time, while externalizing problems increased over time; changes in family functioning were negatively associated with subsequent changes in externalizing problems, and changes in externalizing problems were also negatively associated with subsequent changes in family functioning.

### 4.1 The influence of family function on externalizing problems

Figure 1 shows that the mean slope factor for family functioning among adolescents from low-income families is negative (μ_xs_ = −10.45, *p* < 0.01), while the mean of the slope factor for externalizing problems was positive (μ_ys_ = 9.90, *p* < 0.05). This suggests that both variables exhibited stable longitudinal trends during the follow-up period, supporting Hypothesis 1. This trend aligns with the perspective of family development theory, which posits that the family system undergoes dynamic adjustments in response to the growth of family members, life events, and the external environment (Laszloffy, 2002). As adolescents enter this stage, their need for autonomy increases while their emotional regulation remains underdeveloped, leading to a rise in the frequency and intensity of parent-child conflicts, which may be linked to a decline in family functioning. In low-income households, the convergence of multiple risk factors–such as financial constraints, caregiving pressures, and chronic stress–may be associated with the trend of externalizing problems increasing over time (Xu et al., 2023).

Changes in family function among adolescents from low-income families significantly affect the development of externalizing problems; poor family function leads to the emergence of externalizing problems among adolescents. Conversely, changes in externalizing problems significantly impact family function; the occurrence of externalizing problems also affects the development of family function. This finding supports Hypothesis 2 and validates the reciprocal effects model, aligning with international research such as Kaniousonyte and Zukauskiene (2016) cross-lagged analysis of self-reported parental psychological control and internalizing/externalizing problems among 586 high school students, which identified reciprocal effects between the two. However, that study only identified maternal psychological control as having the strongest influence on adolescents’ externalizing problems. This paper examines changes in adolescents’ externalizing problems from the perspective of overall family care, providing stronger evidence for reciprocal systems theory. Positive family functioning, such as high levels of love and support and positive communication, can effectively reduce adolescents’ externalizing problems (Cambron et al., 2018; Lourdes et al., 2017) and promote their healthy development. Poor family functioning negatively impacts adolescents’ self-regulation systems, increasing their propensity for risk-taking and impulsivity, which in turn leads to externalizing problems and aggressive behaviors (Tian et al., 2018). Children from low-income families, enduring prolonged adverse family environments due to chronic adversity, exhibit externalizing problems more significantly influenced by familial factors (Masarik and Conger, 2017). Economic pressures may lead parents to adopt harsher, inconsistent disciplinary approaches and exhibit emotional detachment during interactions with their children, increasing conflict (Low and Mounts, 2022). Moreover, such stress and scarcity activate the brain’s “survival mode,” impairing cognitive functions and influencing decision-making and behavior (Romer, 2010). Adolescents from low-income families, lacking material support and emotional care, are prone to negative emotions and diminished impulse control. They may seek psychological compensation through maladaptive behaviors, making them more susceptible to rebellious conduct (Portia et al., 2024).

### 4.2 Externalizing problems impact family functioning

Previous research has consistently indicated that high levels of externalizing problems co-occur with increased family conflict, strained parent-child relationships, and reduced family cohesion, creating a negative feedback loop (Wang et al., 2016; Zhao et al., 2017; Luo et al., 2021; Chen et al., 2024; Xia et al., 2025). Behaviors associated with externalizing problems may also increase additional household expenses, further exacerbating economic pressures, and are negatively correlated with improvements in family functioning (Adeniyi, 2022).

This study observed a positive temporal association between adolescents’ externalizing problems and subsequent changes in family functioning in low-income households (γ_xy_ = 0.45, *p* < 0.001); that is, the higher the level of externalizing problems at baseline, the more rapid the subsequent changes in family functioning. This direction of association is inconsistent with most existing literature and is not directly supported by established theories or consistent empirical evidence; therefore, it should not be overinterpreted. This result may reflect the specific nature of the sample context, model specifications, or co-variation patterns among variables and requires replication in further studies. At the same time, changes in externalizing problems were significantly negatively associated with subsequent changes in family functioning (ξ_xy_ = −0.37, *p* < 0.01), suggesting a co-variation between a reduction in externalizing problems and an improvement in family functioning. Combining these two pathways reveals a pattern of mutual co-variation between family functioning and externalizing problems across the longitudinal dimension, consistent with the statistical characteristics of reciprocal effect models and aligned in direction with the findings of relevant longitudinal studies abroad (Kaniousonyte and Zukauskiene, 2016). This study provides four-wave longitudinal evidence from a sample of adolescents in low-income Chinese families from the perspective of overall family functioning, thereby enriching research on the reciprocal dynamics between the family system and adolescent behavioral problems.

### 4.3 Strengths and limitations of this study

Compared with previous domestic studies involving two or three waves of data (Hu et al., 2009; Wang et al., 2022), this study employs four waves of data and a latent change score model, enabling a more detailed depiction of the longitudinal changes in variables and their co-variation pathways, and providing new evidence for understanding the dynamic association between family functioning and externalizing problems among adolescents from low-income families.

This study has several limitations: (1) the model focuses solely on family functioning and externalizing problems, without incorporating internalizing problems or potential mediating mechanisms. Future research could include indicators such as anxiety and depression to further examine multivariate co-variation and chained associations; (2) the data are derived from participants’ self-reported scores, which may be subject to the social desirability effect. Future studies could incorporate multiple perspectives, such as those of parents and teachers, to examine family functioning and externalizing problems; (3) the sample was drawn exclusively from primary and secondary schools in Hunan Province, China, and was limited to low-income families during the targeted poverty alleviation period, resulting in limited generalizability. Future studies could expand the geographic scope, include a wider range of family economic types, and increase sample diversity.

## 5 Conclusion

Family functioning exhibits a significant negative correlation with adolescents’ externalizing problems; the levels of family functioning and externalizing problems change over time; and the dynamic relationship between family functioning and externalizing problems among adolescents from low-income families involves reciprocal effects.

① α represents the constant term parameter, β represents the proportional change parameter; ϕ_x_ and ϕ_y_ denote the autoregressive effects of latent change scores for each variable; γ_yx_ and γ_xy_ denote the level-to-change effects between two variables (i.e., pairing parameters); ξ_yx_ and ξ_xy_ denote the change-to-change effects between two variables; σ_ex_^2^ and σ_ey_^2^ denote residual variances.

## Data Availability

The original contributions presented in this study are included in this article/supplementary material, further inquiries can be directed to the corresponding authors.
